# Prostate Stem Cells in the Development of Benign Prostate Hyperplasia and Prostate Cancer: Emerging Role and Concepts

**DOI:** 10.1155/2013/107954

**Published:** 2013-07-08

**Authors:** Akhilesh Prajapati, Sharad Gupta, Bhavesh Mistry, Sarita Gupta

**Affiliations:** ^1^Department of Biochemistry, Faculty of Science, The Maharaja Sayajirao University of Baroda, Vadodara, Gujarat 390005, India; ^2^Ex-assistant Professor karamsad medical college and Gupta Pathological laboratory, Vadodara, Gujarat 390001, India

## Abstract

Benign Prostate hyperplasia (BPH) and prostate cancer (PCa) are the most common prostatic disorders affecting elderly men. Multiple factors including hormonal imbalance, disruption of cell proliferation, apoptosis, chronic inflammation, and aging are thought to be responsible for the pathophysiology of these diseases. Both BPH and PCa are considered to be arisen from aberrant proliferation of prostate stem cells. Recent studies on BPH and PCa have provided significant evidence for the origin of these diseases from stem cells that share characteristics with normal prostate stem cells. Aberrant changes in prostate stem cell regulatory factors may contribute to the development of BPH or PCa. Understanding these regulatory factors may provide insight into the mechanisms that convert quiescent adult prostate cells into proliferating compartments and lead to BPH or carcinoma. Ultimately, the knowledge of the unique prostate stem or stem-like cells in the pathogenesis and development of hyperplasia will facilitate the development of new therapeutic targets for BPH and PCa. In this review, we address recent progress towards understanding the putative role and complexities of stem cells in the development of BPH and PCa.

## 1. Introduction

Prostate gland is a male accessory reproductive endocrine organ, which expels proteolytic solution in the urethra during ejaculation. In humans, the prostate is located immediately below the base of the bladder surrounding the neck region of the urethra. It is mainly associated with three types of disorders, namely, benign prostate hyperplasia (BPH), prostate cancer (PCa), and prostatitis. BPH and PCa are the most common pathophysiological conditions of prostate gland in elderly men. These diseases already represent significant challenges for health-care systems in most parts of the world. Epidemiologically, BPH is more prevalent in Asian population [1, 2]. Whereas, PCa is more common in the western world [3, 4]. Both the diseases are complex and multifactorial. Factors predisposing to the development of BPH or PCa include hormonal imbalance, oxidative stress, environmental pollutants, inflammation, hereditary, aging, and, more particularly, stromal to epithelial cells crosstalk [5–7]. So far, variety of growth factors and hormonal factors, including androgens and estrogens, has been described in the hyperplastic development of the prostate gland [8–10]. However, the cellular and molecular processes underlying the pathogenesis and development of BPH or PCa are poorly understood.

Stem cells have an extensive capacity to propagate themselves by self-renewal and to differentiate into tissue-specific progeny. It is well know that stem cells are required to maintain and repair tissues throughout the lifetime. The requirement to understand the biology of stem cells derived from the prostate is increasing, as new evidence suggests that BPH and PCa may arise from the stem or stem-like cell compartments [11–13]. This review summarises the biology of prostate stem or stem-like cells and their contribution in pathogenesis and development of BPH and PCa.

## 2. Prostatic Cellular Compartments

The prostate is a hormonally regulated glandular organ whose growth accelerates at sexual maturity due to androgen action on both stromal and epithelial cells [14, 15]. The human prostate is a complex ductal-acinar gland that is divided into three anatomically distinct zones: peripheral, transitional, and central zones, which are surrounded by a dense and continuous fibromuscular stroma [16–18]. BPH, a nonmalignant overgrowth found in older men, mainly, develops in the transitional zone, while PCa arises primarily in the peripheral zone [19].

**Figure 1:**
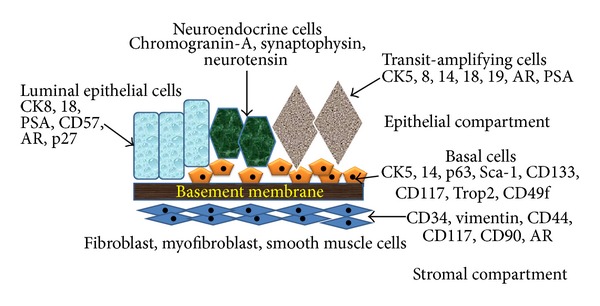
Prostatic cellular compartments and stem cell identity markers. Pictorial representation of different prostatic cells and their respective cellular markers.

At histological level, human prostate contains mainly two types of cells that are called epithelial and stromal cells. The stromal to epithelial ratio in normal prostate of human is 2 : 1 [18, 20]. The epithelial cell layer is composed of four differentiated cell types known as basal, secretory luminal, neuroendocrine (NE), and transit-amplifying (TA) cells that are identified by their morphology, location, and distinct marker expression (Figure 1). The basal cells form a layer of flattened to cuboidal shaped cells above the basement membrane and express p63 (a homolog of the tumor suppressor gene *p53*), Bc1-2 (an anti-apoptotic factor), Cluster designation (CD) 44, hepatocyte growth factor (HGF), and the high molecular weight cytokeratins (CK) 5 and 14. The expression of androgen receptor (AR) is low or undetectable in the basal cells, which makes the basal cells independent of androgens for their survival [21–23]. The luminal cells are the major cell type of the prostate that form a layer of columnar-shaped cells above the basal layer and constitute the exocrine compartment of the prostate, secreting prostate-specific antigen (PSA) and prostatic acid phosphatase (PAP) into the lumen. They are terminally differentiated, androgen dependent, and nonproliferating cells, expressing low molecular weight CK8 and 18, CD57 and p27^Kip1  ^(a cell cycle inhibitor) [22–24] along with high levels of AR. NE cells are rare cells scattered in the basal and luminal layers of the prostate. They are terminally differentiated and androgen-insensitive cells, expressing chromogranin A, synaptophysin, and neuron-specific enolase (NSF) [23, 25, 26]. The NE cells also produce and secrete neuropeptides such as bombesin, calcitonin, and neurotensin that are believed to support epithelial cell growth and differentiation [19, 27, 28]. Additionally, there is a small group of intermediate cells referred to as TA cells that express both basal as well as luminal cell markers (CK5, CK8, CK14, CK18, AR, and PSA) [29–32]. The epithelial layer is surrounded by a stromal layer, which forms a peripheral boundary of the prostate gland. The stromal cell layer consists of several types of cells that include smooth muscle cells (the most abundant cell type in stroma), fibroblasts, and myofibroblasts. Stromal cells express mesenchymal markers like CD34, vimentin, CD44, CD117, and CD90 [33].

## 3. Stem Cell in Normal Prostate

Prostatic epithelium is, structurally and functionally, a highly complex tissue composed of multiple differentiated cell types, including basal, luminal, and neuroendocrine cells, along with small population of relatively undifferentiated cells generally known as “stem cells” that are endowed with self-renewal and differentiation capacities [26]. If the stem cells are key target for mutagenic changes and tumourigenesis in human prostate, we need to understand more about stem cell status in normal prostate tissue. 

As the adult prostate is relatively slow-growing organ with limited cycles of cell proliferation and apoptosis, the possible existence of adult prostate stem cells (PSCs) was controversial for many years. Several investigations based on stem cell models have elegantly defined role of stem cells in cellular turnover and morphogenesis of normal prostate [30, 34]. Evidence for the existence of the stem cells in normal prostate came from the studies which demonstrated that adult rodent prostate can undergo multiple rounds of castration-induced regression and testosterone-induced regrowth [35–37]. Adult PSCs were believed to reside within the basal cell layer because of the ability of the basal cells to survive and undergo regression and regeneration following repeated castration and androgen replacement [38–40]. Adult mouse prostate epithelial cells, when transplanted along with the urogenital sinus mesenchymal cells under the renal capsule, generated normal murine prostate like structures [41]. Prostate glands were also regenerated when dissociated cells were implanted in Matrigel subcutaneously into immunodeficient mice [42]. Studies, including 5-bromo-2-deoxyuridine (BrdU) retention analysis, showed that the enriched population of BrdU-labelled cells possessing stem cell features (quiescent, high proliferation potential) are localized at the proximal region of mouse prostate duct [43] and are programmed to regenerate proximal-distal ductal axis [44]. The proximal region of the prostatic duct is surrounded by a thick band of smooth muscle cells [45] that are known to produce high level of transforming growth factor-beta (TGF-*β*) [46], which is known to play a critical role in maintaining the relative dormancy of the PSCs [47]. Independent study by Burger et al. also identified a candidate population of PSCs in the proximal region of mouse prostatic ducts, using stem cell surface marker known as stem cell antigen 1 (Sca-1, also known as Ly6a) [48]. In addition to high expression of Sca-1, these cells were shown to coexpress integrin *α*6 (CD49f) and Bcl-2. The cells with these properties showed a higher efficiency to generate prostatic tissue in an *in vivo* reconstitution assay [48]. Lawson et al. showed that sorting prostatic cells for CD45(−)CD31(−)Ter119(−)Sca-1(+)CD49f(+) antigenic profile results in a 60-fold enrichment for colony and sphere-forming cells that can self-renew and expand to form spheres for many generations [49]. Leong and colleagues identified CD117 (c-Kit, stem cell factor receptor) as a new marker of a rare adult mouse PSC population that showed all the functional characteristics of stem cells including self-renewal and full differentiation potential. The CD117(+) single stem cell defined by the phenotype Lin(−)Sca-1(+)CD133(+)CD44(+)CD117(+) regenerated functional, secretion-producing prostate after transplantation *in vivo*. Moreover, CD117(+) PSCs showed long-term self renewal capacity after serial isolation and transplantation *in vivo*. CD117 expression was predominantly localized to the proximal region of the mouse prostate and was upregulated after castration-induced prostate involution, consistent with prostate stem cell identity and function [50].

Stem cells in the human prostate have been identified and isolated using the cell surface markers such as integrin *α*2*β*1 [51], CD133 (Prominin-1) [52], and CK6a (cytokeratin 6a) [53]. Based on high expression of *α*2*β*1 integrin, Collins and colleagues identified PSCs in the basal layer and showed that the *α*2*β*1^high^ integrin cells represent ~1% of basal cell population in the human prostate [51]. This selected PSC population was enriched through rapid adherence to the type I collagen and showed higher colony-forming efficiency *in vitro*. Furthermore, when the *α*2*β*1^high^ integrin cells were grafted subcutaneously together with stromal cells in Matrigel into nude mice, they formed prostatic gland structures *in vivo*. Nevertheless, these glandular-like structures, although containing basal cytokeratin positive as well as AR, PAP, and PSA positive cells, lack well-defined basal and luminal organizations [51]. However, recent studies by Missol-Kolka et al. have reported that the overall expression of CD133 in human prostate is not strictly limited to the rare basal stem and progenitor cells, but it is also expressed in some of the secretory luminal cells [54]. Furthermore, it has been shown that CD133 is downregulated in prostate cancer tissues and upregulated in the luminal cells in the vicinity of cancer area. In contrast to the human CD133, the mouse CD133 has been shown to express widely in prostate [54]. Several other surface markers, such as aldehyde dehydrogenase (ALDH), tumor-associated calcium signal transducer 2 (Trop-2), ATP-binding cassette transporter family membrane efflux pump (ABCG2), p63, and CD44, have also been reported for identification and isolation of the PSCs from the prostate tissues of human and mouse [49, 55–60]. Moreover, Trop2(+)CD44(+)CD49f(+) were used as the markers to identify basal stem cells with enhanced prostasphere-forming and tissue-regenerating abilities [61]. Unlike the murine PSCs, the human PSCs are randomly distributed within the basal epithelial layer throughout the acini and ductal regions of the prostate [51, 52]. In addition to the expression of stem-cell-specific markers, different studies have also shown that PSCs express both basal and luminal cell-specific markers in fetal and adult stages of prostate development [13, 22, 31, 62, 63]. Several studies have proposed the existence of different cell compartments based on stem-cell-driven differentiation hierarchical arrangements within the prostate epithelium [24, 29, 30, 64].

In addition to prostate epithelial stem cells, stromal stem cells (SSCs) have also been reported to exist in the prostate, where they are postulated to carry out function of replacing and regenerating local cells that are lost to normal tissue turnover, injury, or aging [65–67]. These subpopulation of SSCs expressed mesenchymal stem cell (MSC) markers such as CD34 and Sca-1, showed a high proliferative activity and ability to differentiate into fibroblastic, myogenic, adipogenic, and osteogenic lineages [68]. Of all these potential lineages, the most characteristic cell type derived from prostate stromal stem cell is fibroblast or smooth muscle cells [68, 69]. Growth factors that have regulatory effects on SSCs include members of TGF-*β* superfamily, the insulin-like growth factors, the fibroblast growth factors, the platelet-derived growth factor, and Wnts [70]. It is believed that the differentiation of stromal stem cells to smooth muscle cells is due to paracrine effects of prostrate epithelial cells, which permanently commit the stromal stem cells to mature into androgen receptor (AR) expressing smooth muscle cells [68].

## 4. Stem Cell in Benign Prostate Hyperplasia (BPH)

BPH is a slow progressive enlargement of the prostate gland which can lead to lower urinary tract symptoms (LUTS) in elderly men. It is characterized by hyperproliferation of epithelial and stromal cells in the transition zone of the prostate gland, which can be observed histopathologically [71]. Despite of its obvious importance as a major health problem, little is known in terms of biological processes that contribute to the development of BPH. To explain the etiology behind the pathogenesis of BPH, several theories, including stem cell, hormonal imbalance, apoptosis, epithelial-mesenchymal transition, embryonic awakening, and inflammation, have been proposed in recent years, and all of them seem to contribute together to some extent in the pathogenesis of BPH [12, 72]. According to stem cell theory, the stem cell population residing in the prostate gland is increased due to abnormal proliferation and apoptosis of stem cells, which may eventually contribute to BPH pathogenesis. Earlier, it was reported by Berry et al. that stem cell population is responsible for prostate gland maintenance [73]. Changes in tissue consistency and cellular hyperplasia are accompanied by downregulation of apoptotic factors and increased level of antiapoptotic factors that decrease the rate of prostatic cell death and, thus, contributing to hyperproliferation of prostatic tissue [74]. It has been reported that stromal to epithelial ratio is altered in BPH, where the ratio increases from 2 : 1 in normal glands to 5 : 1 in BPH [75]. Because stromal hyperproliferative activity is thought to promote the development of BPH, the existence of adult stem cells in the prostate stromal compartment is speculated to expand the stroma in response to stimuli during the pathogenesis of BPH [68]. Lin et al. showed that primary culture of prostate cells from BPH patients possessed many common stem cell markers, including CD30, CD44, CD54, neuronspecific enolase (NSE), CD34, vascular endothelial growth factor receptor-1 (Flt-1), and stem cell factor (SCF, also known as KIT ligand or steel factor) [68]. Compared to CD30, CD44, CD54, and NSE, the CD34, Flt-1, and SCF markers were expressed at low level. These stem cells were negative for CD11b, stem cell antigen-1 (SCA-1), SH2, AA4.1, and c-Kit. Furthermore, among this stem cell population only a fraction (5%) of the stem cells was positive for CD133 [68]. Although the origin of these stem cells is not known, the CD49(+)CD54(+)NSE(+)SCF(+) cell marker profile of these cells suggests that they are in a lineage closely related to MSCs. The stem cell population with the above profile possessed ability to differentiate or transdifferentiate into myogenic, adipogenic, and osteogenic lineages [68, 76]. Ceder et al. reported the possible existence of prostate stromal stem/progenitor cells in the adult human prostate [76]. This stromal population expressed vimentin (a mesenchymal marker), CD133, c-Kit, and SCF, with expression profiles similar to those observed in the Cajal cells of gastrointestinal tract, which represent a subset of stem cell-like cells. Several studies have identified c-Kit-expressing interstitial cells in the stromal compartment of human prostate [77–79]. Altered patterns of c-Kit expression have been reported in benign lesions of prostate and breast tissues [80, 81]. It has been shown that the c-Kit expression and number of c-Kit(+) interstitial cells were significantly higher in BPH than those of the normal prostate. Furthermore, it has been suggested that c-Kit regulates cell proliferation in prostate and plays a crucial role in the pathophysiology of BPH via altering the expression of JAK2 and STAT1 [77].

Stem cells from the BPH samples expressing CD49f, CD44, or CD133 markers have been shown to possess monolayer- and spheroid-colony-forming ability, where the highest (98%) recovery of colony-forming cells (CFCs) was achieved by CD49f(+) cells as compared to CD44(+) (17%) or CD133(+) (3%) cells [82]. These CFCs showed the capacity to undergo clonal proliferation, generates branching ductal structures, and they expressed both basal and luminal lineage markers. Further characterization of CD49f(+) cells revealed that they are comprised of two cell types: CK5(+) basal epithelial cells and CD31(+) endothelial cells [82]. Sca-1- and CD34-expressing cells isolated from BPH tissue showed a high proliferative capacity and increased plasticity, as these cells were able to differentiate into fibroblastic, myogenic, adipogenic and osteogenic lineages, similar to that of MSCs [68, 83]. Furthermore, Burger and colleagues found that cells with high Sca-1 expression had considerably more growth potential, and proliferative capabilities than cells expressing low or no Sca-1 antigen [48]. Expression of pluripotency markers such as *Oct4A*, *Sox2*, *c-Myc*, and *Klf4* might represent a stemness-specific gene signature. A very recent study has demonstrated a relatively high expression of stemness-associated genes, including *Oct4A*, *Sox2*, *c-Myc*, *Nanog*, and *Klf4*, in BPH as compared to normal prostate tissue [84]. Thus, several studies have revealed the presence of stem cells that express pluripotency-associated markers and are hyperproliferative and capable of differentiation into different cell lineages within the hyperplastic prostate tissue. The presence of these high proliferative and plastic stem cells in the BPH tissue samples suggests that BPH could occur as a result of changes in the stem cell properties that could ultimately give rise to a clonal expansion of cell populations.

## 5. Stem Cell in Prostate Cancer (PCa)

PCa is the most prevalent and is the second most frequently diagnosed cancer and sixth leading cause of cancer-related deaths among men in the world [85]. Its etiology, although not clear, is partly attributed to multigenic and epigenetic mechanisms and the heterogeneous nature of this disease [4, 86–88]. Gleason and others described that when the transition of normal gland into adenocarcinoma of prostate takes place, its normal histological structure is disrupted and results in abnormal proliferation of the glandular structure, destruction of basement membrane, and progressive loss of basal cells (<1%) [87, 89]. In addition, AR(+) luminal cells increase and contribute in bulk of prostate mass (>99%) in PCa [90]. It is hypothesised that prostate cancer arises from AR(+) luminal cells and dramatic loss of basal cells. To support this hypothesis several investigations have been conducted [4, 91–93]. In addition, mouse basal population expressing Lin(−)Sca-1(+)CD49f^high^ cells can differentiate into luminal cells in xenograft [49]. Lin(−)Sca-1(+)CD49f^high^ cells from a Pten−/− mouse model display cancer stem cells phenotypes, which gave rise to adenocarcinoma after transplantation [94]. It has been reported that basal cells are the possible cells of prostate cancer origin [95]. When Goldstein et al., especially injected the mixture of urogenital sinus mesenchyme (UGSM) with human prostate basal (expressing CD49f^high^ and Trop2^high^) or luminal cells (expressing CD49f^low^ and Trop2^high^) into the subcutaneous space of immunodeficient NOD(−)SCID(−)IL(−)2Rg−/− mice, only basal cells formed prostatic duct after 16 week, whereas no prostatic duct or adenocarcinoma developed when using luminal cells [91, 95]. Luminal derived grafts lack epithelial structures and mimicked transplantation of UGSM cell alone [95]. Collins et al. reported basal cancer stem cells isolated from human prostate cancer biopsies expressing Cd44(+), *α*2*β*1^high^, and Cd133(+) and cell surface markers were of self renewal *in vitro* [96]. ALDH^high^ is another marker used for cancer stem cells in human prostate cancer cell lines. Cells expressing ALDH^high^ *α*2(+)/*α*6(+)/*α*v(+)-integrin CD44(+) showed increased tumourigenicity and metastasis *in vivo* and enhanced invasiveness *in vitro* [97]. Prostate cancer stem cells isolated from LNCaP and DU145 cell lines also showed expression of CD44(+), *α*2*β*1^high^, and CD133(+) markers [98, 99]. In addition, CD44(+) population isolated from xenograft human tumour and cell lines displayed high tumour initiating ability and metastasis *in vitro* [100]. Recently, Rajasekhar and his group isolated a small cell population expressing TRA-1-60(+)CD151(+)CD166(+) markers that displayed stem cell like features with increased NF-kB signalling along with basal cell markers, and this recapitulates the cellular hierarchy of the tumour origin from basal cells [101].

 Over all data from several investigators indicated that origin of prostate cancer can be from basal stem cell population, which expresses CD44(+), *α*2*β*1^high^, CD133(+), ALDH^high^, and other normal basal stem cell markers.

## 6. Stem Cell Niche and Plasticity

Stem cells are localized in a defined microenvironment, which is known as their “niche.” The main function of a niche probably is to provide specific factors necessary for the maintenance of the stem cell properties via a combination of intracellular and intercellular signalling. These factors include a complex array of growth factors, cytokines, chemokines, and adhesive molecules known to be capable of altering the balance between proliferation, differentiation, and quiescence in stem cell populations [102, 103]. One can probably assume that this is equally true for prostatic stem cells as it is for other stem cell populations.

PSCs reside in niche areas within the basal layer of the epithelial compartment at a low percentage of approximately 0.5–1% [34]. PSCs population in the prostate undergoes a series of phenotype changes. Specifically, the basal SCs do not express the AR or the p63 protein. They have extended proliferative potential by slow cycling. According to these studies, it is postulated that, in addition to the reserve stem-cell population, there is a “TA” cell type, which is characterized by the expression of p63, as well as other basal markers such as CK5 and 14, Jagged-1, and Notch-1 [64, 104, 105]. A TA cell does not express AR protein and it is dependent, for proliferation, but not for survival, on andromedans secreted by stromal cells [105]. Under normal conditions a PSC is slow cycling in that it divides occasionally, undergoing asymmetric division to give rise to a new PSC along with a more differentiated TA daughter cell. TA cell undergoes a limited number of rapidly amplifying cell division cycles to increase the cell population derived from a single PSC before leaving the proliferative compartment to produce intermediate cell [106]. This intermediate cell expresses both epithelial specific (CK5 and 14) and luminal specific (CK8 and 18) cytokines, AR mRNA (but not protein), and prostate stem cell antigen (PSCA) [105, 107]. As an intermediate cell migrates through the basal layer, it differentiates into various terminally differentiated cell lineages of prostate epithelium. 

## 7. Is BPH/PCa a Stem Cells Disease?

**Figure 2:**
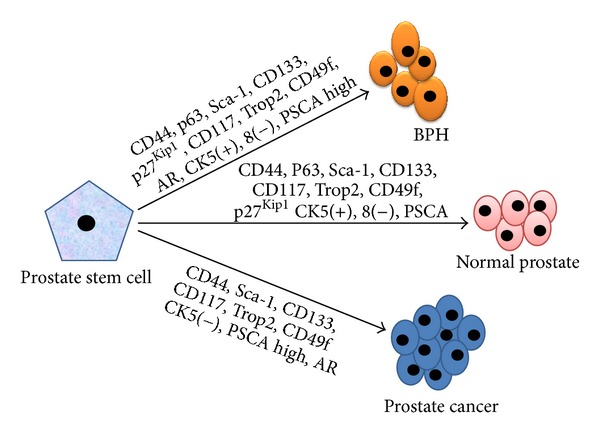
Cellular identity of stem cells in prostate. Stem cell model of normal tissue renewal, BPH and PCa.

Numerous investigators demonstrated presence of stem cell in prostate tissue by using various high-end techniques that may contribute to local invasive to metastatic disease in human and research animals. In normal tissue-development, homeostasis is maintained by differentiation of stem cells and programmed cells death in regular cell cycle. This mechanism is established through interactions with tissue specific environmental factors such as growth factors and steroid hormones. Many signalling molecules and factors involvement have been reported in stem cell self-renewal and implication in cancer stem cells (CSCs) regulation (Figure 2).

Although the precise role of stem cells in tumourigenesis is still in debate, it is widely accepted that cancers can arise from normal stem cells which may accumulate mutation, genetic changes, and molecular pathway alterations that disrupt self-renewal control capacity (Table 1). It has been reported that, in prostate, putative stem/progenitor cells can reside in CK5(+) 8(−) basal cells. A diagnostic feature of human prostate cancer is the loss of basal cells [108], indicating cancer origin cells as basal cells. In BPH, CD133(+) cells expressed genes related to undifferentiated cells such as TDGF1 (teratocarcinoma-derived growth factor 1) and targets of the Wnt and Hedgehog developmental pathways, whereas CD133(−) cells showed upregulation of genes related to proliferation and metabolism. In cancer, CD133(+) cells specifically displayed more TA population phenotype with increased metabolic activity and proliferation, possibly explaining the transition from a relatively quiescent state to an active growing tumour phenotype. This reflects that CD133 isolates from benign and malignant tissues show biologically distinct characteristics [109]. CSCs exploit many of the signal pathways such as notch, hedgehog- and TGF-*β*, which play, important role in proliferation and differentiation in prostate stem cell [110, 111]. The sonic hedgehog signalling element receptor PTCH1 and glioma-associated oncogene homolog-1 (GL1) transcription factor were especially reported to be colocalized with p63 basal marker in BPH and PCa cells, expressing CD44/CK8/14. This suggests that hedgehog pathway may induce differentiation of prostate stem/progenitor cells into CD44(+)/P63(+/−) hyperplasia basal cells [112]. Other studies on DNA damage and proliferation markers p27^Kip1^, cyclin D3, and Ki-67, revealed interesting findings. It has been shown that p27^Kip1^ is significantly upregulated in BPH, whereas it is downregulated in PCa. In addition to downregulation of p27^Kip1^, there is also up regulation of Ki-67 and cyclin D3 in PCa [113]. 

Several lines of evidence have been indicated that CSCs exhibit both stem cells and cancer cells characteristics. CSCs have the ability to form tumors when transplanted into an animal host. CSCs can be distinguished from other cells within the tumor by cell division and alterations in their gene expression profile [114].

Advanced prostate cancer is androgen independent and basal cells can be phenotypically identified in the majority of metastases [115]. Studies from several investigators revealed that tumor-initiating cells are negative for AR and p63 and expressed the stem cell markers Oct-4, Nanog, Sox-2, Nestin, CD44, CD133, and CD117. Moreover, Sca-1-positive cells having the ability with prostate-regeneration activity, showed evidence of a basal and luminal lineage [96, 100, 116, 117]. Gu et al. demonstrated human telomerase reverse transcriptase-(hTERT-) positive epithelial cells could regenerate tumor in mice that resembled the original tumor in patients [118]. These finding may be indicative of CSC role in prostate cancer.

The growing understanding of the prostate stem cell biology provides the rationale for acute approaches. But without a clear definition of stem cells in normal prostate and BPH/PCa, it is difficult to determine whether the cancer cell of origin in prostate is a stem cell, multipotent progenitor/TA cells, or a more differentiated progeny. Nonetheless, evidence exists that the cellular origin can include both basal and luminal cells.

## 8. Conclusion 

The prostate stem cells are a key role player in prostate tumourigenesis and enlargement disorders. But their precise role in disease pathogenesis remains unknown. The prostate stromal and epithelial compartments and their reciprocal paracrine and autocrine interactions are crucial regulators of prostatic tissue homeostasis. The combination of the prostatic cell surface markers, such as Sca-1, CD133, p63, and CD49f, can aid in the identification of prostate stem cell populations. However, a prostate-specific stem cell marker has yet to be identified. The study of CSCs is still in its early stages. No standard treatments have yet been developed as a result of research on CSCs. The isolation and characterization of epithelial, stromal stem cells and cancer stem cells in the prostate will lead to understanding normal stem cells and CSCs activity to identify new strategies for the control of prostate diseases without harming normal cells milieu.

## Figures and Tables

**Table 1:** Molecular alterations in BPH and PCa.

Factors	Normal prostate	BPH	PCa
Prostate-specific factors			
5 *α* reductase	Normal	Upregulated	Upregulated
Androgen receptor (AR)	Normal	Upregulated	Upregulated
AR coactivator	Normal	Upregulated	Upregulated
Androgen corepressor	Normal	Upregulated	Upregulated
PSA level in serum	(0–4 ng/mL)	(2–8 ng/mL)	(4–10 ng/mL)

Growth factors	FGF-2,7,9 IGF 1,2 IGFBP-2	FGF 1,2,9 IGF-2 high IGFBP-3	FGF-1,2,6,8 IGF-1 high IGFBP-2 high IGFBP-3 high

NE cells	Normal	Number decrease	Number increase

Luminal cell factors	Vimentin	Vimentin increase	Vimentin over exp
	Intracellular space normal	Intracellular space increase	Intracellular space decrease
	PMSA normal	PMSA decrease	PMSA increase

Basal cells	Present	Present	Absent

Stromal cell factor	Fibroblast content normal	Fibroblast content increase	Fibroblast content increase
	NMMHC	NMMHC increase	NMMHC
	Elastin	Elastin decrease	Elastin increase
	SMMHC	SMMHC decrease	SMMHC decrease

Stem cell markers	CD44, P63, Sca-1, CD133, CD117, Trop2, CD49f, p27^Kip1^, CK5(+), 8(−), PSCA	CD44, p63, Sca-1, CD133, p27^Kip1^, CD117, Trop2, CD49f, AR, CK5(+), 8(−), PSCA high	CD44, Sca-1, CD133, CD117, Trop2, CD49f, CK5(−), PSCA high, AR
